# Factors Impacting Quality of Life of Caregivers of Cancer Patients in the Sub-Himalayan Region: A Cross-Sectional Study

**DOI:** 10.7759/cureus.35168

**Published:** 2023-02-19

**Authors:** Kaalindi Singh, Ekta Dogra, Kapil M Pal, Ratti R Negi, Ranveer Vardhan, Ritu Sharma, Kalpana Katoch

**Affiliations:** 1 Department of Radiotherapy, Shri Lal Bahadur Shastri Government Medical College and Hospital, Mandi, IND; 2 Department of Community Medicine, Shri Lal Bahadur Shastri Government Medical College and Hospital, Mandi, IND; 3 Pediatrics, Rohilkhand Medical College and Hospital, Bareilly, IND

**Keywords:** factors, quality of life, cancer, burden, caregiver

## Abstract

Background

Caregivers of cancer patients experience excessive emotional and financial stress.

Objective

To determine the quality of life (QOL) of caregivers of cancer patients and factors affecting it in caregivers attending the OPD of a governmental tertiary care cancer center in the sub-Himalayan region.

Methods

A cross-sectional observational study was used. A pre-validated caregiver quality of life (CQOL) questionnaire was completed by consenting caregivers of 96 outpatient attendees.

Results

The mean total QOL scores were higher in attendants of subjects who did not undergo surgery versus those who underwent surgery (p-value: 0.04) and in those who received 0-5 versus >5 chemotherapy cycles (p-value: 0.015). On subdomain analyses, the burden was significantly greater in caregivers of patients who did not undergo surgery (p-value: 0.02) and had a higher Eastern Cooperative Oncology Group (ECOG) scale (p-value: 0.03). Disruptiveness was significantly higher in married individuals (p-value: 0.01) and those aged between 36 and 60 years (p-value: 0.003). Positive adaptation was worse in caregivers with another family member suffering from chronic illness (p-value: 0.041) and in professional and skilled workers (p-value: 0.03). The greater financial burden was experienced by caregivers belonging to lower socioeconomic status (p-value: 0.03). On putting these factors in the regression model, the non-specific domain of 0-5 cycles had poor CQOL, i.e., higher scores. The burden score was more in ECOG stages 3 and 4 in comparison with stages 1 and 2. Married individuals had significant disruption. Individuals aged between 36 and 60 years perceived more disruption in their lives. A more financial impact was observed on the lower middle and lower class compared to the upper and upper middle class.

Conclusion

Caregivers of cancer patients experience significant stress and burden. Counseling and social and financial support to caregivers may help improve CQOL.

## Introduction

The burden of cancer in India as well as worldwide, is progressively increasing. As per GLOBOCAN 2020 [1], 13,24,413 new cancer patients were diagnosed in India alone in 2020, and the prevalence of cancer was 27,20,251 cases. Cancer treatment is prolonged, tedious, and often expensive, leaving the family emotionally exhausted and financially drained [2,3]. Thus, the diagnosis of cancer has a significant impact not only on the well-being and quality of life (QOL) of the afflicted individual but the entire family. Many studies examining the QOL among cancer patients have been reported in the literature [4-9]. However, caregiver QOL (CQOL) often remains neglected. Studies examining the QOL among caregivers in our country are limited, and none have been reported from the sub-Himalayan region. As the QOL may be affected by regional and socio-cultural variations, in this study, we undertook to determine the QOL and factors influencing it in caregivers of cancer patients in the sub-Himalayan region.

## Materials and methods

Study design

Appropriate ethical clearance from the Institutional Review Board Ethical Committee was obtained before the expedition of the study. A cross-sectional survey-based study design was used. Caregivers of cancer patients reporting to OPD of the tertiary care cancer center (TCCC), SLBS Government Medical College, Mandi, Himachal Pradesh, were enrolled. 

Study participants

Caregivers of 96 patients reporting to our outpatient unit for chemotherapy or follow-up were enrolled in a consecutive order. The inclusion criteria for enrollment were 1) age >18 years, 2) related or actively involved in the care of patients with histologically proven malignancy, and 3) capable of understanding and completing the questionnaire on their own or with some assistance. Caregivers incapable of understanding and completing the questionnaire were excluded.

Measurement tools

A pre-validated CQOL questionnaire [10] was used for the collection of QOL data. This questionnaire had 27 questions for assessing four major domains of caregiver well-being and eight items that do not pertain to any specific domain. The four major domains included burden (10 items), disruptiveness (7 items), positive adaptation (7 items), and financial concerns (3 items). In the burden subdomain, caregivers were asked about their fear, sadness, and mental stress about their patient's health condition. In disruptiveness, aspects pertaining to difficulties caregivers experience due to changes in their daily routine and priorities were addressed. In positive adaptation, patients talked about their discouragement about the future and inability to manage their loved one's pain. A higher score indicated a poorer adaptation. The financial domain had questions related to the caregiver's economic difficulties. The last non-specified domain comprised questions pertaining to caregivers' interpersonal relationships, sexuality, and spirituality. The questionnaire had answers on a Likert scale from 0 to 4, from which the caregivers select the most appropriate answer. Higher scores are indicative of a poorer QOL, with a maximum score of 140.

Data collection

Caregivers were taken to a private area to explain the purpose of the study, and valid consent was taken. Questionnaires were provided in Hindi and English for the caregivers to fill in. The Hindi version of the questionnaire was derived by the forward and backward translation method, and validity was established using Cronbach's alpha. The investigators clarified any doubts pertaining to the questions and the study. The investigators also filled up a proforma containing the patient and caregiver demographics and treatment factors.

Data analysis

The collected data were tabulated on the computer using Microsoft Office 2010, Excel package. Cronbach's alpha, KMO test, and Bartlett's Test of Sphericity were done to assess the reliability of the questionnaire. Further, the rotated component matrix was calculated through the varimax method, wherein each statement factor or loading was evaluated.

For comparing the means of two groups (unpaired, unequal variance), a two-tailed unpaired Student's t-test was done, and ANOVA was done when comparing the means of three or more groups. Rejection criteria for the null hypothesis were taken as p<0.05. In step 2, variables that were found to be significant in univariate were put in the logistic regression model. Here dependent variables were demographic factors like age, annual family income, education, etc., and the independent variable was the CQOL score.

## Results

A total of 96 caregivers consented to be part of the study. The mean age of caregivers was 39±12 years, with the majority being males. The detailed demographic variables studied in the participants and the total mean CQOL scores are depicted in Table 1.

**Table 1:** Sociodemographic and treatment variables of study participants. CQOL-Q: Caregiver Quality of Life Questionnaire; CQOL: Caregiver Quality of Life; KP scale: Kuppuswammy scale; ECOG: Eastern Cooperative Oncology Group.

Variable	N (%)	Missing Values N (%)	Total Mean CQOL scores	P-value
Sex of attendant				
Female	35 (36.5)		58.54±23.4	0.8
Male	61 (63.5)	0	60.11±22.3	
Marital status				
Married	78 (81.2)		60±23.6	0.7
Unmarried	18 (18.8)	0	57.6±18.0	
Age of caregiver				
18-35 years	51 (53.2)		56.2±20.3	0.1
36-60 years	38 (39.6)		65.2±25.2	
61-100 years	7 (7.3)	0	52.9±20.2	
Age of patient				
18-35 years	2 (2.1)		79.5±13.4	0.4
36-60 years	49 (51.1)		57.9±24.1	
61-100 years	45 (46.9)	0	60.5±22.6	
Socio-economic status (KP scale 2020) of patient				
Upper and upper middle class	25 (26)		54.6±23.1	0.4
Middle class	20 (20.8)		59.3±21.5	
Lower middle and middle class	50 (52.1)	1 (1.04)	61.9±23.5	
Profession of caregiver				
Homemaker	22 (22.9)		53.1±19.4	0.051
Semiskilled worker or student	57 (59.4)		64.0±23.4	
Professional or skilled worker	12 (12.5)	5 (5.2)	47.7±19.7	
Educational status of caregiver				
Illiterate	3 (3.2)		56.7±19.3	0.7
Primary till secondary	54 (56.3)		61.9±22.5	
Diploma till postgraduate	37 (38.5)	2 (2.1)	56.2±23.7	
Stage of disease of patient				
Stage I	12 (12.5)		67.3±29.2	0.5
Stage II	13 (13.5)		54.5±23.7	
Stage III	28 (29.1)		57.6±23.9	
Stage IV	41 (42.7)	2 (2.1)	60.1±19.9	
ECOG staging of patient				
0-1	77 (80.2)		57.9±22.9	0.1
3-Feb	19 (19.8)	0	66.4±20.5	
Stage of treatment of patient				
Radical	49 (51)		58.8±24.5	0.3
Palliative	38 (39.5)		63.3±19.5	
Follow-up	6 (6.25)	3 (3.1)	49.5±26.2	
Relationship of caregiver				
Parents and siblings	3 (3.1)		48.7±20.3	0.7
Children and grand-children	55 (57.3)		60.3±19.8	
Spouse and In-laws	38 (39.6)	0	59.3±26.6	
Habitat				
Rural	18 (18.8)		60.5±22.5	0.4
Urban	75 (78.1)	3 (3.1)	55.0±24.7	
Surgery of patient				
Yes	60 (62.5)		56.1±23.6	0.04
No	36 (37.5)	0	65.3±20.0	
Radiotherapy of patient				
Yes	43 (44.8)		60.5±23.7	0.7
No	51 (53.1)	2 (2.1)	59.3±22.1	
Total duration of illness of patient				
Less than 6 months	47 (49)		59.2±21.1	0.9
More than 6 months	49 (51)	0	59.9±24.2	
Comorbidities among caregivers				
Diabetes mellitus				
Yes	6 (6.3)		55.5±22.9	0.7
No	90 (93.8)	0	59.8±22.7	
Hypertension				
Yes	5 (5.2)		56.2±27.6	
No	90 (93.8)	1 (1.04)	59.7±22.5	0.7
Family structure				
Nuclear	23 (23.9)		62.0±24.9	0.5
Joint	72 (75)	1 (1.04)	58.8±21.9	
Any other chronic illness in family of caretaker				
Yes	17 (17.7)		67.2±20.4	0.1
No	78 (81.2)	1 (1.04)	57.9±22.9	
Habits among caregivers				
Alcohol intake				
Yes	75 (78.1)		59.1±22.5	0.7
No	21 (21.9)	0	61.1±23.6	
Smoking				
Yes	10 (10.4)		66±19.2	0.4
No	85 (88.5)	1 (1.04)	59.3±22.6	
Presently employed				
Yes	52 (54.2)		61.9±24.5	0.3
No	43 (44.8)	1 (1.04)	56.5±20.2	
Enrollment in governmemnt schemes				
Yes	57 (59.4)		59.1±25.3	0.9
No	38 (39.4)	1 (1.04)	60.1±18.6	
Any other health insurance of patient				
Yes	4 (4.2)		48.5±27.2	0.4
No	78 (81.2)	14 (14.6)	59.3±23.2	
Number of chemotherapy cycles				
0-5 cycles received	77 (80)		63.2+20.1	0.015
> 5 cycles received	19 (20)	0	48.8+26.8	
BMI of caregiver				
< 18.4	6 (6.3)		63.5±26.4	0.5
18.5-22.9	40 (41.7)		55.3±19.9	
23.0-27.4	34 (35.4)		64.1±24.9	
≥ 27.5	16 (16.7)	0	56.5±22.6	

Table 2 depicts the mean scores of subdomains of CQOL, mainly disruptiveness, financial concerns, burden, positive adaptation, and non-specific domains.

**Table 2:** Subdomain wise scores of CQOL-Q. CQOL-Q: Caregiver Quality of Life Questionnaire; CQOL: Caregiver Quality of Life.

Scores of different domains	Mean ± SD
Total CQOL Scores	59.5 ± 22.6
Burden CQOL Domain	16.1 ± 09.5
Positive Adaptation Domain	09.7 ± 05.8
Disruptive Domain	08.1 ± 05.3
Financial Domain	03.8 ± 03.5
Non specific Domain	23.5 ± 05.8

The CQOL domains across various demographic factors were studied. The total mean score of CQOL was significantly lower if the patient had undergone surgery in comparison to no surgery (56.1±23.6 and 65.2±20.0, respectively, with a p-value of 0.04 and 5% CI: 18.1-0.2). Also, the mean CQOL-C scores in caregivers of patients receiving 0-5 cycles of chemotherapy were higher as compared to those who received more than five cycles, i.e., 63.1±20.7 and 48.8±26.8 respectively (p-value: 0.015, CI: 2.9-25.9). The mean burden score was significantly lower amongst the caregivers whose patient had undergone surgery than those who had not (14.3±9.2 and 19±9.3, respectively, with a p-value of 0.02). The mean burden score was significantly higher in caregivers of ECOG 2 and 3 patients (20.2±8.4) compared to ECOG 0 and 1 (15.0±9.5), with p-value being 0.03 and CI: 0.6-9.9. Higher mean scores in positive adaptation were present if any other family member was suffering from chronic illness (12.3±3.6) when compared with no family member suffering from any chronic illness (9.1±6.1), p-value being 0.041 and CI: 0.1-6.2. Mean scores of the positive adaptation domain were significantly higher in professional and skilled workers (11.0±6.1) in comparison with the semi-skilled workers and students (6.3±5.1) (p-value: 0.03). Married individuals experienced significantly greater disruption (8.8±5.4) than unmarried (5.2 ± 3.7) (p-value: 0.01 and CI: 0.9-6.2). Individuals aged between 36 and 60 years had more disruption (10.3±5.4) than individuals aged between 18 and 35 years (p-value: 0.003 and CI: -6.2- -1.1). The financial domain of CQOL scores had a significant relationship amongst various socioeconomic classes as per the Kuppuswammy scale 2020, with lower financial impact in the upper and upper middle vs. lower middle and lower class. The mean scores were 3.7 ±2.7 and 8 ± 2.9, respectively, with a p-value of 0.03 and CI: 0.2-10.4. In the non-specified domain, the mean score had a significant relationship with the number of chemotherapy cycles, with patients receiving 0-5 cycles having a higher score of 25.0±4.9 than those receiving more than five cycles, i.e., 18.9 ± 6.2 (p-value <0.01 with CI: 8.8-3.4). On doing logistic regression in step 2, none of the factors studied in univariate analysis were found to be significant in terms of total mean CQOL scores. In the non-specific domain, 0-5 cycles had poor CQOL, i.e., higher scores. The burden score was more in ECOG stages 3 and 4 in comparison with stages 1 and 2. Married individuals had significant disruption. Individuals aged between 36 and 60 years perceived more disruption in their lives. A more financial impact was observed on the lower middle and lower class compared to the upper and upper middle class.

Factor analysis and reliability

Cronbach's alpha of the caregiver quality of life questionnaire (CQOLQ) was 0.885 for 35 statements with a KMO value of 0.796. Furthermore, Bartlett's Test of Sphericity value was less than 0.05. The three test values of CQOLQ were found reliable and significant for conducting factor analysis. Further descriptive statistics and Cronbach's alpha were calculated to evaluate the mean and SD, and reliability of the 35 statements. A positive but moderate correlation was observed between the statements, and their reliability was greater than the tolerance limit of 0.70. Hence it was inferred that the collected data was reliable and fit to evaluate factors from these 35 statements.

Factor loading

Total variances have been explained by the principal component matrix, wherein components whose Eigenvalue is greater than one have been considered. Overall, 87.1% of the data variable could be explained via these five factors or associates. The rotated component matrix was calculated through the Varimax method, wherein factor loading was evaluated for each statement. As per the data, the five primary factors or components were as follows: burden (factor loading=95.6%, α=88.0%), others (factor loading=94.0%, α=88.1%), disruptiveness (factor loading=91.0%, α=88.2%), financial concerns (factor loading=85.8%, α=88.8%) and lastly, positive adaptation (factor loading=84.0%, α=88.5%).

## Discussion

Due to differences in the health setup, family structure, culture, and socioeconomic status, the factors impacting the QOL differ significantly from region to region [11-13]. Therefore, we undertook to determine factors that impact the QOL of caregivers in the sub-Himalayan region of India. Caregivers of patients with ECOG 2-3 experienced a higher burden than better ECOG patients, which is understandable as patients with worse ECOG are more dependent on caregivers for their activities of daily living (ADL). Socioeconomic status significantly impacted the financial domain of the QOL, with lower classes having worse QOL scores in the financial domain. Married individuals and individuals between the ages of 36 and 60 years experienced greater disruptiveness in their routines. This could be because this subgroup of individuals have greater family responsibilities and, in most cases, are the breadwinners. They need to make more adjustments in their routine to accommodate caregiving activities. Thus, caring for cancer patients can cause them greater disruption than younger or unmarried individuals who do not have as many responsibilities yet. Lastly, the remaining non-specific domain was affected by the number of chemotherapy cycles. Patients who received 0-5 cycles had a higher score than those who received >5 cycles. The possible causes could be greater apprehension during the initiation of chemotherapy, followed by easing into the treatment process with time. In one study by Huang CY et al., greater adaptation has been suggested among caregivers with a longer duration of patient illness [14]. Factors like gender, relationship with the patient, stage of disease, background, family structure, treatment intent, caregiver educational status, comorbidities among caregivers and patients, and enrollment in health schemes did not significantly impact the CQOL. Regarding factor loading of sub-domains, the burden had the highest factor loading. The second important factor was the non-specific domain which comprises spirituality, interpersonal relationships, and sexuality. The third factor was disruption, followed by financial loss, and lastly, positive adaptation.

In a study by Lukhmana et al [15], on quality of life of caregivers of cancer patients, married status, daycare treatments, unemployed cancer patients and nuclear family structure lead to increased burden among caregivers.

In a similar study from AIIMS, New Delhi, Mishra S et al. [16] assessed caregiver's burden and QOL in caregivers of cancer patients on chemotherapy. Zarit Burden Interview (ZBI) and WHOQOL BREF questionnaire were used to assess burden and QOL. The burden was significantly more among females and unemployed caregivers. In our study, unemployed caregivers fared worse in the financial domain, but there was no gender difference in the burden experienced by caregivers. In a recent study by Kulkarni SS et al. [17] conducted in rural India, the mean CQOL-C score was 44.15 ± 17.24 (CI: 41-47.3). This was less than the scores of caregivers in our region, indicating better QOL than our caregiver population. Recurrent, metastatic and hematological malignancies had a significant impact on the QOL of caregivers. In our study, the disease stage or treatment intent did not directly impact CQOL. Other factors like the number of chemotherapy cycles, and another person in the family with chronic illness, which seemed to have an impact on QOL in our study, were not evaluated in the above studies. Girgis A et al. [18] were first to document caregiver burden. The stress experienced by caregivers can be reduced by counseling and addressing the queries of caregivers regarding the patient stage, prognosis, and homecare required during treatment [19,20]. Counselors, trained palliative care nurses, volunteers, and community support groups may provide caregiver respite [21,22]. In our setup, where these are unavailable in all centers, routine OPD contact should be optimized for providing information and counseling to caregivers. Other coping strategies like meditation, yoga, and exercise should be encouraged. To reduce the financial burden, several healthcare schemes have been introduced by the government for cancer patients. At the same time, treatment schedules that are equally effective but require fewer hospital visits and are more affordable can considerably reduce the financial toxicity to caregivers. The limitations of this study are, being a hospital-based survey, caregivers who were interviewed might not have been the actual caregivers of cancer patients. Only caregivers with better understanding were interviewed which may also cause a bias in results. The caregivers attending only the chemotherapy unit were enrolled, which could lead to bias in results. The correlation of type of surgery with QOL was not obtained in patients who underwent surgery. Despite these limitations, this is the first such study in the sub-Himalayan population and paves the way for improving the QOL among caregivers in this region.

## Conclusions

Caregivers of cancer patients are silent sufferers. Their needs are rarely addressed in our health setup and society. In this study, treatment factors like the number of chemotherapy cycles, socioeconomic status, caregiver age, and marital status were found to significantly impact CQOL and its subdomains. With an increasing incidence of cancer, the caregiver burden is bound to increase and become a public health problem. Timely measures may minimize the impact of this adversity in the community.
